# Adapting a Patient-Reported Outcome Measure to Digital Outpatient Specialist Health Care Services for Type 1 Diabetes: User Involvement Study

**DOI:** 10.2196/38678

**Published:** 2022-11-15

**Authors:** Heidi Holmen, Tone Singstad, Lis Ribu, Annesofie Lunde Jensen, Nina Mickelson Weldingh, Astrid Torbjørnsen

**Affiliations:** 1 Department of Nursing and Health Promotion Oslo Metropolitan University Oslo Norway; 2 The Intervention Centre Oslo University Hospital Oslo Norway; 3 Endocrinology Outpatient Service Akershus University Hospital Lørenskog Norway; 4 Centre for Senior Citizen Staff Oslo Metropolitan University Oslo Norway; 5 Department of Clinical Medicine Faculty of Health Aarhus University Aarhus Denmark; 6 Steno Diabetes Centre Aarhus Aarhus University Hospital Aarhus Denmark; 7 ResCenPI - Research Centre for Patient Involvement Aarhus University & the Central Denmark Region Aarhus Denmark; 8 Department of Research Support Service Akershus University Hospital Lørenskog Norway

**Keywords:** patient-reported outcome measures, user involvement, type 1 diabetes, digital interventions

## Abstract

**Background:**

Diabetes self-management is crucial for patients with type 1 diabetes, and digital services can support their self-management and facilitate flexible follow-up. The potential of using digital patient-reported outcome (PRO) measures in routine outpatient care is not fully used owing to a lack of adapted PRO measures.

**Objective:**

This study presents the process of identifying and adapting a digital PRO measure for use in clinical diabetes practice and describes the preferred item topics of the adapted PRO measure, as reported by patients and diabetes specialist nurses.

**Methods:**

With the involvement of patients, diabetes specialist nurses, management, and researchers, we hosted a series of workshops and 2 dialogue conferences. Scoping searches to identify relevant PRO measures formed the foundation for the process. An in-person dialogue conference was conducted with diabetes specialist nurses as participants, and a digital dialogue conference was conducted with patients with type 1 diabetes as participants. A diabetes-specific PRO measure was translated and adapted to our digital platform. Notes and summaries from the dialogue conferences were imported into NVivo (QSR International) and thematically analyzed as a single combined data set.

**Results:**

The thematic analysis of the 2 dialogue conferences aimed to explore the views of patients with type 1 diabetes and diabetes specialist nurses on the outcomes necessary to measure. An overarching theme, *Ensuring that the PRO measure captures the patients’ needs precisely and accurately, in a way that facilitates care and communication with health care personnel*, was identified and supported with data from both the patients and diabetes specialist nurses. This theme contained four categories: *The need for explanatory text after questions to ensure understanding and accurate response*, *Capturing individual needs in standardized questions, getting to the heart of the patient’s problem*, and *The questions increase patient reflection*.

**Conclusions:**

We successfully conducted an iterative process that identified a PRO measure aligned with the topics raised by the diabetes specialist nurses. Similarly, the patients found the PRO measure to be relevant and one that was addressing their needs. Only minor adjustments were necessary when programming the PRO measure in the digital platform. Our management, patients, and diabetes specialist nurses had a valuable impact on the results. User involvement facilitated a specific focus on the clinical requests to be met by PRO measures and how they must be adapted to local and digital platforms. Overall, this has facilitated the current implementation of the adapted digital PRO measure.

## Introduction

### Background

Approximately 10% of the 463 million people with diabetes have type 1 [1], placing an increasing strain on limited resources in the health care service. Diabetes self-management is crucial to live a healthy life, attending to the following treatment cornerstones: what one eats, how one exercises, and medication and blood glucose to prevent complications [2]. In addition to microvascular and macrovascular complications, diabetes can be associated with psychological problems such as diabetes distress [3], anxiety [4], and depression [5]. Although self-management is a continuous affair [6], the knowledge needed to self-manage can increase stress. For example, as the patients become more knowledgeable, their fear of late complications and long-term consequences may compromise quality of life [7]. Altogether, the complex demands of diabetes are a burden on the patient and health care services. Patients with type 1 diabetes are usually offered diabetes follow-up organized through diabetes teams in hospital outpatient services [8,9]. Attitudes toward diabetes care have evolved from compliance thinking toward the support of patients active in their diabetes self-management [2]. In addition, the advancement of medical equipment for insulin delivery and glucose management has evolved with insulin pumps and continuous glucose monitoring systems, which forms new demands to the delivery of health services. However, the organization of health services, with fixed consultations at suboptimal or inconvenient times for patients, often hinders this evolution in routine care [2]. Failure to attend scheduled outpatient appointments is an increasing problem among patients with type 1 diabetes and is associated with both suboptimal diabetes care and nonsustainable economic loss [10]. Combining the focus on objective clinical parameters such as hemoglobin A1c (HbA_1c_) level, time in range, and similar parameters with subjective experiences of the symptom and treatment burden of patients could reduce the psychosocial strain of type 1 diabetes [2-5,7].

A subjective or self-reported measure is often labeled as *patient-reported outcome* (PRO), defined as “any report of the patient’s health condition that comes directly from the patient, without interpretation of the patient’s response by a clinician or anyone else” [11]. PRO measures are measures or methods used to gather these patient reports. This is complementary to objective measures such as blood pressure, height, weight, or various blood measures. The use of PRO measures is suggested to support communication between patients and health care personnel (HCP) and to individualize the provided health care, for instance, in an outpatient consultation [12-14].

Despite the recent clinical uptake of PRO measures, there is a paucity of systematic reviews of PRO measures in diabetes care, and the evidence base largely comprises primary studies. Studies investigating the use of PRO measures to guide outpatient consultations for patients with type 1 diabetes indicates both its usefulness for guiding consultations [15] and its perceived relevance [16]. In addition, from the perspective of HCP, organizational aspects such as time, training, and facilities of a preconsultation PRO measure must be accounted for in any clinical use of PRO measures [17]. Furthermore, a generic PRO system called AmbuFlex has been a pioneer in flexible follow-up and the use of PRO measures to assess the need for consultations [18,19]. Patients in AmbuFlex are sent a digital PRO measure 2 weeks before a scheduled consultation to assess their prioritized need for follow-up. The timing, duration, and content of consultations can be tailored to the individual needs and priorities of the patients. The goal is to get patients engaged in their own self-management, treatment, and need for health care as a support for chronic illness self-management [20]. HCP using AmbuFlex refer to both advantages and disadvantages of digital PRO measures [21]. AmbuFlex has recently been evaluated for patients with type 1 diabetes (DiabetesFlex version 1 containing 45 items), where it maintained safe diabetes management, improved well-being, and decreased the need for face-to-face visits [22,23]. Complementary to these findings, an analysis of preconsultation PRO measures shows that HCP, on one hand, need training to interpret patient responses [24]. In contrast, patients value the opportunity to add free-text details to their answers [24]. Furthermore, the subjective PRO measures must complement the objective clinical measures of diabetes self-management [25]. PRO measures in routine diabetes visits can be feasible and acceptable among patients and HCP [26].

Thus, the increased use of PRO measures in clinical practice can facilitate user involvement; however, research is scarce [12]. A recent review highlighted the importance of involving users and other stakeholders in the development of PRO measures and subsequent implementation and transformation of clinical services [14]. It adds to previous literature emphasizing how both patients’ and HCP’s preferences for relevant measures should be considered [27]. There is more to a life with diabetes than HbA_1c_, and psychosocial outcomes are crucial as they are often associated with glycemic control [28]. Pragmatic studies investigating opportunities for the development and implementation of more user-oriented health services have real potential to improve services [14,23].

Implementation of PRO measures in routine care for type 1 diabetes complies well with the overarching responsibility for diabetes self-management within complex diabetes care. Health services need to move toward a service built upon the needs of and requests from patients and not those from the health service to enhance the support of patient self-management. Implementing PRO measures can facilitate this change, and if they are developed and tailored together with users, we believe that actual change can be seen.

### Aims and Objectives

We present the process of identifying and adapting a digital PRO measure for use in clinical diabetes practice and describe the preferred item topics of the adapted PRO measure, as reported by patients and diabetes specialist nurses.

## Methods

### Dialogue Conference Methodology

Dialogue conferences are useful for sharing and gathering reflections that will have consequences for the invited participants. The suggested aim of dialogue conferences is to act as an arena for communication, where a broad range of participants can be mobilized [29]. In our study, the relevant participants were patients, diabetes specialist nurses, management, and researchers. Our intention was to conduct a series of dialogue conferences with all participants at the same time. Owing to the COVID-19 pandemic, we adapted our methodological process and conducted both in-person and digital conferences. Thus, the participants were invited on separate occasions; diabetes specialist nurses were invited to an in-person session, and patients were invited to a separate digital session. The digital sessions were hosted on Whereby (Whereby) [30], the platform used for treatment consultations and thus familiar to the patients. The research team and management of this study worked iteratively with the material throughout the study. The overall aim of the dialogue conferences was to enable all users to be active partners to facilitate the adaptation of a digital PRO measure in their health service.

### Overall Design

**Figure 1:**
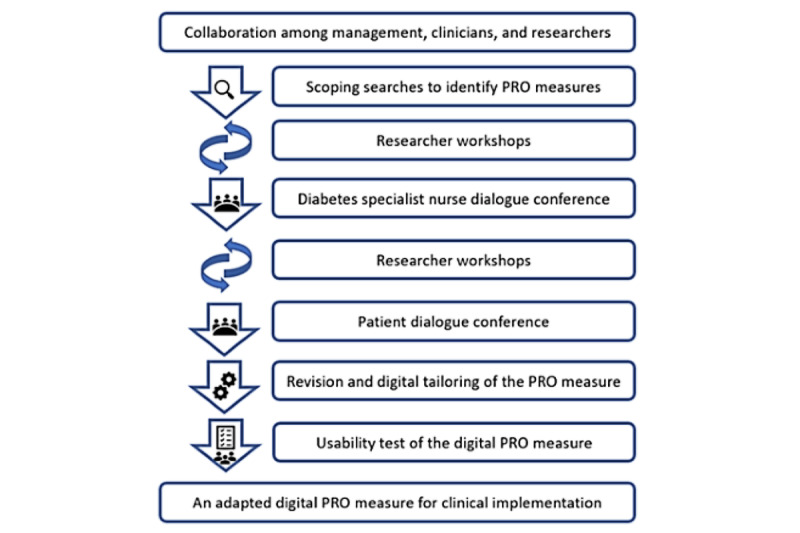
Process to identify, evaluate and adapt a set of patient-reported outcome measures for digital clinical implementation.

This study was founded in a collaboration among management, clinicians, and researchers, with a common aim of implementing a digital PRO measure with clinical relevance for patients with type 1 diabetes at a specialized outpatient service. The stepwise methodological outline for this study is presented in Figure 1.

### Participants

In total, four groups of users were included in the current process: management, researchers, diabetes specialist nurses, and patients. All invited participants accepted their invitation.

Management included 2 individuals, 1 (50%) trained as a diabetes specialist nurse holding a leadership position and 1 (50%) trained as a nurse holding a strategic position in organization development. Both had a Master’s degree.

The researchers comprised a group affiliated with a university and a group affiliated with a university hospital in full-time or part-time clinical positions. All but one researcher were women (4/5, 80%). Of the 5 researchers, 2 (40%) were formerly trained as nurses, 1 (20%) was formerly trained as a diabetes specialist nurse, and 2 (40%) were physicians. All of them (5/5, 100%) had a clinical background in their field and a PhD.

At the outpatient clinic, 5 diabetes specialist nurses are employed. All (5/5, 100%) were invited to participate in the dialogue conference, but only 80% (4/5) of them could attend. Thus, 4 female diabetes nurses with a range of 5 to 15 years of experience with patients with diabetes participated in the study.

Patient participants were recruited through convenience sampling and a personal invitation by a diabetes specialist nurse at the outpatient clinic. The patients comprised a group of 8 adults (n=2, 25% men and n=6, 75% women), with age ranging from early twenties to late seventies. Their ethnic backgrounds were from Europe and Asia. Their duration of diabetes ranged from 6 to 60 years, and they had a variety of experiences with treatment regimes, including multiple daily injections of insulin, daily injections of long-acting insulins, and insulin infusion pumps with a tube and those without a tube (such as the tubeless OmniPod). For self-monitoring of blood glucose, the participants shared their experiences with finger prick and various systems for continuous glucose monitoring and flash glucose monitoring.

### Scoping Searches to Identify Relevant PRO Measures

To identify relevant PRO measures, we conducted systematic and exploratory searches to obtain an overview [31]. An example of the search is available in Multimedia Appendix 1. Overall, two researchers (HH and AT) independently assessed the citations and included those of relevance before they were discussed with the research team. Data extraction was conducted according to an a priori set of criteria including author and year of publication, name of the instrument, author and year of the development or validation of the instrument, aim of the instrument, number of items, availability of the PRO measure in Norwegian or another Scandinavian language, whether it had open access or was licensed, any experiences from use in clinical practice or research, and other comments regarding the instrument.

### Analysis of the Dialogue Conferences

The dialogue conferences were not recorded, but notes were taken consecutively by 2 designated researchers at the dialogue conference with the diabetes specialist nurses, and 3 designated researchers took notes at the dialogue conference with the patients. These notes were imported into NVivo (QSR International) for storage and analysis. Thematic analysis, as described by Braun and Clarke [32,33], was applied as it is a widely adopted method for qualitative analysis and suggested to be an accessible approach for researchers. It consists of six phases: (1) familiarizing yourself with your data, (2) generating initial codes, (3) searching for themes, (4) reviewing themes, (5) defining and naming themes, and (6) producing the report [32]. Notes from the abovementioned dialogue conferences provided the data for thematic analysis. The initial categories were discussed by the research group until consensus was reached, and the joint discussion led to the overall theme reflecting the content and meaning of the categories.

### Usability Testing of the Digital PRO Measure and Digital Platform

The patient participants, management, and researchers were granted access to a test platform in which the tailored digital PRO measure was programed, with the opportunity to test the usability of the software system and items. They were asked to evaluate the PRO measure items, content, readability, interface, and usability and to provide feedback through the chat function in the software program. These findings are presented descriptively in the *Results* section.

### Ethics Approval

The institutional local data protection officer approved the project (19/06920). All participants received written and oral information regarding the project and its aim, and informed consent was obtained from all participants.

## Results

### Results From the Scoping Searches

We identified 68 unique PRO measures aiming to address diabetes-specific outcomes. In a research workshop, the list of 68 PRO measures was reduced to an initial shortlist of 10 (15%) and further reduced to 5 (7%) PRO measures relevant for type 1 diabetes [22,34-37]. The main reason for exclusion was PRO measures addressing outcomes irrelevant for type 1 diabetes. The 7% (5/68) of the shortlisted PRO measures are listed in Table 1 along with their characteristics. These were presented at the diabetes specialist nurses dialogue conference.

**Table 1:** Shortlist of PRO^a^ measures (n=5; alphabetically ordered).

Study (author, year)	Country	Name of the PRO measure	Aim of the PRO measure	Number of items	Available languages	Licensed	Used in clinical practice
Jensen et al [22], 2020	Denmark	DiabetesFlex version 1	To assess psychosocial and physical symptoms and prepare and focus the conversation between the patient and health care personnel	Maximum=45 (items are responsive, and questions are reduced or added based on the responses)	Danish	No	Yes
Brod et al [35], 2006	Denmark	Diabetes Medication Satisfaction (Diab-Med-Sat)	To measure diabetes treatment satisfaction, applicable to a wide range of diabetes therapies	23	English, Danish, Norwegian, and many more	Yes	Yes
Gold et al [37], 1994	United Kingdom	Gold	To assess hypoglycemia awareness	1	English, Norwegian, and many more	No	Yes
Carlton et al [36], 2017	United Kingdom	Health and Self-management in Diabetes–10 (HASMID)	To measure the impact of self-management in diabetes	8	English	No	No
Alvarado-Martel et al [34], 2017	Spain	Vida con Diabetes tipo 1 (ViDa1)	To measure health-related quality of life in patients with type 1 diabetes	34	Spanish and English	No	No

^a^PRO: patient-reported outcome.

### Results From the Initial Researcher Workshops

In preparation for the dialogue conferences and summary work after the conferences, a series of workshops was conducted among the management and researchers. In preparation for the first series of workshops, the findings from the scoping searches were systematized by HH and AT in a Microsoft Excel file containing the name of the instrument, author and year of publication, where the instrument was identified, whether it was used in Norway, availability in Norwegian, whether it was used in research or clinically, aim of the instrument, and who made the original development. The extracted data were sent to the research team. In total, 2 videoconference workshops were conducted, aiming to reduce the number of relevant PRO measures and form a shortlist of instruments before the diabetes specialist nurse dialogue conference. Between these 2 workshops, the researchers and management reviewed a list of identified diabetes-specific PRO measures and noted their comments on the PRO measure and its characteristics as extracted. Each member commented according to their individual thoughts of relevance as a preparation for joint discussion. A new series of workshops among the researchers was conducted to summarize the data gathered in the first dialogue conference and to prepare PRO measures to discuss with patients during a new dialogue conference.

### Summary of Diabetes Specialist Nurses Dialogue Conference to Explore and Evaluate PRO Measures

The dialogue conference with the diabetes specialist nurses opened with the opportunity to freely discuss their views on diabetes care today, whether and how digital service may be useful, opportunities with electronic health records, and how these may provide inspiration for future services.

Post-it notes were used to scribble ideas for specific items or questions that they regarded as crucial to gather information on. The diabetes specialist nurses were concerned that the information gathered using a PRO measure is designed for self-reporting by the patient. Where patients are responsible to self-report their current situation and needs, the nurses were afraid that they would miss out on an opportunity to detect something that the patient would have left out from their self-report—intentionally or unintentionally. A nurse simply said, *“*we need the obvious,” without adding details to what the obvious are. They initially discussed details regarding glycemic control more than other aspects of diabetes; however, after some time, the discussion shifted. After discussing the details regarding hypoglycemic events, stability or lack thereof in HbA_1c_ level, treatment regime, technical equipment, injection technique, time in range, and any fluctuations in blood glucose that patients cannot explain, there seemed to be a gradual shift toward the more psychosocial aspects of diabetes. The diabetes specialist nurses emphasized that there is more to life than diabetes, and, sometimes, the treatment regime should be adjusted because there is just too much going on. Obtaining this knowledge through items with fixed responses may be challenging, and free text was suggested as valuable.

When the initial open discussion ended, a short break was taken before the 5 PRO measures presented in Table 1 were discussed. Overall, the diabetes specialist nurses reported that all the presented PRO measures had some relevant items. They were familiar with the 1-item PRO measure addressing hypoglycemia in plain language—“Do you know when your hypos are commencing?” answered on a 7-point Likert scale ranging from *1* (*always aware*) to *7* (*never aware*), developed by Gold et al [37], and found it useful. However, in addition to this item on hypoglycemia awareness, the overall impression was of the negative framing of the items, asking about how troublesome or problematic the aspects of diabetes treatment were. Having diabetes is obviously life-changing, but not something that can be removed. Asking about what is normal was also perceived as problematic by the diabetes specialist nurses because *normal* can mean many things. Furthermore, they questioned the relevance of items on mood and discussed the importance of asking patients about parts of their treatment that they could offer some guidance on. The majority of the diabetes specialist nurses were in favor of the diabetes-specific questions. Overall, items on hypoglycemia, support, control, and social activities were considered as important. The DiabetesFlex questionnaire version 1 [22] covered much of what had already been discussed as necessary and was perceived as relevant and more positively framed; however, some cultural adaptation from Danish to Norwegian would increase its relevance.

### Results From the Researcher Workshops Between the Dialogue Conferences

After the nurses’ dialogue conference, the researchers met to summarize and plan for the next step. Relevant topics, themes, and items were collected based on the input and notes from the diabetes specialist nurses, and 56 item topics were identified (Multimedia Appendix 1). These item topics were presented to the management and researchers in a workshop, and as a result we found that Diabetes Flex [22] covered most of these item topics in a favorable manner. The decision to go forth with the Diabetes Flex was presented and discussed in the clinic, and mutual agreement was reached among the researchers and clinical staff to concentrate on the items in the DiabetesFlex. Being a result of many years of collaboration among researchers, patients, and HCP [22,23], it was particularly regarded as clinically relevant. At this point, researcher ALJ was not participating in these discussions or the decision to move forward with a Norwegian adaptation of DiabetesFlex; rather, she was consulted afterward. As the research team behind DiabetesFlex had revised version 1 (45 items) and version 2 was available, we concentrated on the latter with 69 items. The overlap with our identified items and desired framing of questions served as a confirmation of relevance (Multimedia Appendix 1).

The authors, TS and NMW, drafted the first translation of the DiabetesFlex questionnaire version 2, and AT, LR, and HH reviewed and revised the translation. Items already translated into Norwegian, such as the World Health Organization–5 (WHO-5), were identified and used. The final translation of the remaining items that were not previously translated was discussed with researchers and clinicians before it was presented to ALJ, who participated in the Danish development and testing of the DiabetesFlex.

### Summary of Patient Dialogue Conference to Explore and Evaluate PRO Measures

The Norwegian translation of the DiabetesFlex questionnaire was distributed to patients who had signed up for a digital dialogue conference. The patients received two files: one file with the mandatory annual consultation questionnaire containing 66 items and one with the optional consultation questionnaire containing 37 items. There is an overlap between the 2 questionnaires (Multimedia Appendix 1). The digital dialogue conference was hosted with 8 patients, 4 members of the research team, and 1 observer from the learning and mastery center at the hospital. In short, the patients felt that the items covered their needs and were adequately framed and easy to understand, but they were numerous and seemed somewhat too much to handle at first glance. The patients also added some reflections regarding life in general. A patient said the following:

If life is hard at the moment, it is not necessarily a bad idea to discuss this.

However, another patient suggested that diabetes should be the only focus of the questions.

### Results From Thematic Analysis of Dialogue Conferences

#### Overview

In the following section, the combined thematic analysis of the 2 dialogue conferences is presented, along with a descriptive summary of the usability test of the adapted digital PRO measure. The aim of the 2 dialogue conferences was to explore the outcomes necessary to measure in type 1 diabetes and to assess a set of PRO measures. Both the diabetes specialist nurses and patients referred to some tacit knowledge when they discussed the relevance of questions, what to add, and what to delete. The thematic analysis [32] of the notes taken during the 2 conferences resulted in 4 categories (Textbox 1). These reflect the shared opinions among the patients and nurses on what a clinical PRO measure for diabetes must address to be relevant for all stakeholders. Through discussions among the researchers, an overall theme emerged from the 4 categories: *Ensuring that the PRO measure captures the patients’ needs precisely and accurately, in a way that facilitates care and communication with HCP*.

The theme, *Ensuring that the PRO measure captures the patients’ needs precisely and accurately, in a way that facilitates care and communication with HCP*, has four categories: *Need for explanatory text after questions to ensure understanding and accurate response*, *Capturing individual needs in standardized questions, getting to the heart of the patient’s problem*, and *The questions increase patient reflection*, shedding light on the content and purpose as experienced by diabetes specialist nurses and patients with diabetes (Textbox 1).

**Theme and categories obtained from the thematic analysis of the dialogue conferences.**

**Theme**

- Ensuring that the patient-reported outcome measure captures the patients’ needs precisely and accurately, in a way that facilitates care and communication with health care personnel

**Categories**

- Need for explanatory text after questions to ensure understanding and accurate response
- Capturing individual needs in standardized questions
- Getting to the heart of the patient’s problem
- The questions increase patient reflection

#### Need for Explanatory Text After Questions to Ensure Understanding and Accurate Response

Both the diabetes specialist nurses and patients highlighted the need for questions that were immediately understood and the need to explain in detail about what was asked for. An example could be to explain where the patients can find their HbA_1c_ level or what is meant by *time in range*. Explanatory text could also appear after the patient had given their response, to provide some immediate guidance to self-manage or link to help pages or other relevant sources. For example, if they indicated severe symptoms, such as chest pain or trouble breathing, text with information regarding emergency health services should appear, emphasizing the need to contact these services.

Hypoglycemia was raised as a frequent and important topic to address. However, when a patient feels a hypoglycemic episode commencing and the blood glucose values differ from one person to another. To address the burden of hypoglycemia, the patient participants suggested to simply ask if hypoglycemia was experienced as a problem, and thus to replace phrases such as “How often do you experience hypoglycemia with a blood glucose reading at 3.9 mmol/mol or lower?”

Some questions should have had some context added to their explanation, as they could be challenging to interpret and answer. Some patients had comorbidities that affected their health more than diabetes, and some clarification was requested, such as for questions asking to assess pain that should specify whether a response should be related to their general health or their health related to type 1 diabetes.

#### Capturing Individual Needs in Standardized Questions

The diabetes specialist nurses highlighted their need to apply a holistic care perspective. A nurse said the following:

We cannot help if we cannot see the whole person.

Individual patients have unique needs, preferences, and beliefs, and a nurse mentioned an example of patients being on each side of a scale, where one may be totally ignorant of their disease, whereas the other is extremely frightened of late complications and fear that they will lose their eyesight tomorrow. Patients suggested that the opportunity to add their own text after some standardized questions could allow for their individual needs to be explained:

If you really want to know how we are doing, you have to add a free-text field.

Another patient also suggested the need for free text related to situations in which the given response interval would not fit their response, for example, where they wanted to answer between 2 boxes.

Similarly, the diabetes specialist nurses discussed letting patients add information in free-text fields, but they were concerned about whether patients were able to recognize their needs and put these needs into words. They were skeptical about self-reporting as they had experiences with faulty self-reports, both intentionally by providing blood glucose readings that seemed better than they actually were or unintentionally, such as recall bias, where patients suggested that they had nocturnal hypoglycemic episodes, whereas it had only been the previous night.

#### Getting to the Heart of the Patient’s Problem

The diabetes specialist nurses were not concerned about the number of questions. Especially, if there were boxes to tick or intervals to indicate, for example, units of insulin taken, the questionnaire would be rather quick to complete. They worried more about whether patients were able to indicate their problems by using a free-text field. Again, patients suggested that free-text fields had to be an option if HCP were to get to the heart of their problems. The patients felt that the questionnaire they had been presented was slightly lengthy, with 69 items, but they agreed that the topics were relevant and necessary. A patient also asked whether some of the questions could be responsive; that is, if you indicate having symptoms, a list will appear, but if you indicate no symptoms, you are spared from having to look at the list of symptoms.

Patients also acknowledged the questions asking about their *worry* or how often they think about diabetes-related problems or challenges. These topics were appreciated by the patients more than those asking about their quality of life. A patient said the following:

Some of these questions...being worried is one of the things that makes us tired. Very relevant question, this must be included. Very important question.

#### The Questions Increase Patient Reflection

Patients stated through the dialogue conference that their thoughts had begun to wander as they read the questions they had been sent in preparation. A patient said that the questions made her reflect upon her daily life and the choices she made. Another patient added some arguments and said that it had increased the motivation to obtain more knowledge and to obtain an HbA_1c_ level measurement more often. Furthermore, the questions regarding symptoms and late complications also prompted reflections among the patients, and, although it was brutal, a patient said the following:

This became a bit serious question, very important for reflection, to think through. This is the problematic side of the disease. Can stand exactly as it does.

Similarly, the diabetes specialist nurses suggested that some topics were not necessarily important for them to know about, such as whether patients needed their prescriptions filled or whether they had attended their eye examination, but they could act as a reminder for the patients.

### Usability Testing of the Digital PRO Measure and Platform

Patient participants (8/8, 100%), HCP (5/5, 100%), management (2/2, 100%), and researchers (5/5, 100%) were granted access to a test platform for usability testing. Input was accounted for in the process of tailoring the DiabetesFlex questionnaire to fit in a Norwegian digital platform. Responses were provided by 75% (6/8) of the invited patients who had participated in the previous dialogue conference, 60% (3/5) of the researchers, 100% (2/2) of the management representatives, and 100% (5/5) of the HCP. Responses included comments in favor of the free-text fields, that the items covered all necessary aspects of a life with type 1 diabetes, and that the inclusion of items covering areas they normally would not address in a traditional consultation was valued. More technical responses targeted the platform and covered feedback on items that obviously should not be mandatory, such as problems with erections that had to be answered by female respondents because of a default function in the digital system making all questions mandatory. Furthermore, if one missed a question, they would not automatically be sent back to that specific question; rather, they had to skim through all the questions to find the blank one and give their response.

### Revisions of the PRO Measure

The PRO measure was revised according to feedback from the patients before the usability testing and after the usability testing, before clinical implementation. Most revisions were the addition of explanatory text to the items. For instance, in the first version, the patients were asked to report their HbA_1c_ level, and in the revised version, text was added to explain which value they should report: “What is your last HbA_1c_ (Last HbA_1c_ measured at any doctor’s office within the last 6 weeks).” Similarly, when asking for the mean blood glucose level for the past 2 weeks, explanatory text was added to guide the patients to find and report the right value. Finally, we added an item asking the patients to specify the date on which the HbA_1c_ level was determined.

## Discussion

### Principal Findings

In this study, we intended to obtain deep insight into patients’ and diabetes specialist nurses’ requirements regarding PRO measures in diabetes type 1. Moreover, we aimed to explore how these requirements would transfer to a digital platform. Specifically, we wanted to identify and adapt a PRO measure that would meet the requirements and be suitable for digital reporting and interpretation.

The results of our iterative process led to the identification of an overall theme describing the demands of patients and diabetes specialist nurses: *Ensuring that the PRO measure captures the patients’ needs precisely and accurately, in a way that facilitates care and communication with HCP*. The patients and diabetes specialist nurses showed great consistency when they spoke about their needs and demands. Both groups acknowledged the importance of glycemic control and the need to report details of blood glucose, hypoglycemia, and insulin regime. Both groups talked about the need to report how things really are, beyond numbers. Similarly, both groups mentioned problematic aspects of standardized questions, particularly relevant because diabetes type 1 can be very different from person to person [2-7]. Consistent with previous studies [15,17,24,25], our participants highlighted the importance of asking understandable questions, explaining them in detail if necessary, and adding open-text fields for patients to elaborate and aid an increased understanding of the patient’s situation. Neither group reflected on the lack of age-adjusted PRO measures. However, making the items responsive of preceding reports were mentioned as favorable. This allows for some adjustments in the items the patient is presented with, based on their needs or characteristics.

The diabetes specialist nurses were concerned about the patients’ ability to put their needs into written words. In contrast, the patients were concerned about not having the opportunity to use their own words to describe a problem. This highlights the challenge of how needs can be communicated through a format that is different from conversation. However, if a PRO measure report is outside the anticipated thresholds, a dialogue with HCP will have to occur and the patient will have an opportunity to elaborate [23]. However, both groups regarded PRO measures as relevant in prompting reflection among patients. Standardized items can address topics that the patient would not think about as relevant for their diabetes [24-26]. In this way, patients can become more knowledgeable about their disease, its signs and symptoms, and steps necessary for self-management through their digital self-report [20]. Although digital platforms for diabetes self-management support have been used for some time [38,39], there are few studies investigating how digital PRO measures can be used to assess and prioritize the need for contact with diabetes outpatient services [40,41]. Furthermore, the challenges associated with digital PRO measures in clinical care are the same in digital mode as when using paper. Our diabetes specialist nurses had some concerns about the risks of using digital PRO measures to assess and prioritize patients’ need for follow-up. Thus, the PRO measures have to ask for the most relevant aspects of the disease, treatment, and self-management [24-26]. In addition, the risk of self-report bias is not to be underestimated, both for overreporting and underreporting of symptoms [42]. Similarly, the threat of recall bias must be accounted for in the decisions on how frequently a PRO measure should be distributed to the patient. These factors can greatly influence the decisions made by HCP [24,26]. However, besides the risk of not asking for essential information, neither diabetes specialist nurses nor patients reflected on the practical interpretation of the patient report and the consequences the interpretation has for the patient treatment. The standardization of items may facilitate easy interpretation by the diabetes specialist nurses and explain why free text is seldom used in clinical PRO measures [43]. In addition, standardized questions may seem more relevant, prompting relevant disease actions among both HCP and patients, thus facilitating self-management and further use of digital PRO measures. Future studies should investigate how the PRO measures can prompt self-management and their associations with interpretations and treatment by HCP.

Most PRO measures that we identified were developed to act as outcomes in intervention studies and not to tailor clinical patient care. This lack of clinically relevant PRO measures that are easy to administer has been discussed previously [14]. Thus, the diabetes specialist nurses did not immediately see the clinical value of all the PRO measures. For example, the nurses worried that the PRO measure would reveal or address problematic areas in which they could not offer any treatment or follow-up. Furthermore, some of the PRO measures that we identified were found by the diabetes specialist nurses to be negatively framed, long, asking irrelevant questions, and not considering any subjective input (eg, through open-text fields). Digital platforms can, to an extent, offer more dynamic PRO measures that are tailored to the patient’s characteristics, for example, their age, treatment, or challenges. However, more studies have to investigate the reliability of such dynamic alternations. With the current pandemic and potential shortage of HCP, new digital and dynamic methods to assess patient needs are warranted, and only few PRO measures relevant for clinical application in diabetes exist.

In our study, we aimed to adapt a PRO measure for digital, clinical use. When developing such new processes of care for implementation, both patient and HCP perspectives must be addressed [15,17,21,24,26]. Often, lack of personal motivation or engagement and low quality of the digital platforms are hindering implementation [44]. Furthermore, ease of use has been described as an important facilitator for digital interventions, and lack of exposure to or knowledge of digital interventions is considered as the most important barrier [45]. Through our early involvement of stakeholders from all levels, we have tried to prevent these barriers. Although user involvement can be time consuming and resource demanding, it is valuable as it addresses all stakeholder perspectives in the early development [25,45]. Our 2 dialogue conferences and the inclusion of users in the final usability test have contributed to the likelihood of implementing a relevant and useful PRO measure in a digital platform that is useful and easy to engage with [45]. Interestingly, we found that patient engagement was easy to pursue using a digital platform for communication and interaction, and the number of patients willing to participate was higher than that for our in-person meetings. The extent of this positive impact on recruitment should be investigated in future studies to facilitate increased user involvement.

### Limitations

Our study has some limitations, particularly related to our user participants. We had no family members and other HCP besides diabetes specialist nurses, and we did not have the opportunity to gather all users in 1 meeting at the same time, which may have reduced the potential for valuable input, as we did not manage to facilitate joint discussions among use groups. However, we had a heterogeneous sample of patient representatives, ensuring a width in our input. Furthermore, we included the patient users later in the process than preferred. We acknowledge that early involvement of patients could have affected our results. We could have pursued the inclusion of more patient groups, but we were able to accomplish tailoring and adapting a PRO measure to a digital platform through valuable feedback. Our patients can be biased and more engaged in digital platforms than the average patient, and thus more capable of understanding the questions. During the recruitment of patients, we strived to obtain a heterogeneous group in terms of age, gender, and ethnicity. However, selection bias is a well-known challenge, and we acknowledge that our users can be a more well-functioning group, consistent with previous studies [24,25]. Our study was conducted in a Scandinavian country, adapting a Scandinavian PRO measure, and the PRO measures’ relevance for other areas has not been addressed. Future studies should pursue adaptation in other countries to assess its relevance. Regarding the inclusion of other HCP, we consider diabetes specialist nurses as the most important group, as they are the first responders and those who are closest to patients through the current organizational structure. Thus, we believe that their engagement and response are of utmost importance, with specific impacts on the adapted PRO measure.

The chosen PRO measure contains a combination of previously validated measures and items, in addition to some self-developed ones. These have not been validated in combination, and we did not pursue to validate the adapted PRO measure in this study. This would be important to address in future studies.

### Conclusions

We have shown how a process aiming to identify and adapt a PRO measure into a digital platform for clinical use in a diabetes outpatient clinic has been conducted. The involvement of management, patients, and diabetes specialist nurses had a valuable impact on our results. This process has been crucial in facilitating the forthcoming implementation success, as our stakeholders have contributed to a digital PRO measure that addresses relevant topics and is used in a user-friendly digital platform. Allowing user involvement using digital platforms was also found to be a favorable method, as it increased attendance beyond expectations. We anticipate positive outcome from our digital PRO measure because we ensured user involvement of highly invested stakeholders, thus ensuring the relevance of our PRO measure.

## Appendix

Multimedia Appendix 1 Identified items and overlaps with the original DiabetesFlexTM questionnaire.

## Abbreviations

HbA_1c_: hemoglobin A_1c_
HCP: health care personnel
PRO: patient-reported outcome
WHO-5: World Health Organization–5

## Data Availability

The Norwegian version of the DiabetesFlex questionnaire can be made available upon request to ALJ. The English version 1 is published elsewhere [23].
