# Supplementary material for: Adapting a Patient-Reported Outcome Measure to Digital Outpatient Specialist Health Care Services for Type 1 Diabetes: User Involvement Study
Source: JMIR Hum Factors. 2022 Nov 15;9(4):e38678. doi: 10.2196/38678 (PMC9709667; doi:10.2196/38678)
Supplement: Multimedia Appendix 1 [file humanfactors_v9i4e38678_app1.docx]

## Appendix 1. Identified items and overlaps with the original DiabetesFlex^TM^ questionnaire.

|  | **Item topics generated in the dialogue conference with the diabetes specialist nurses, N=56 items** | **Item topics covered in the original DiabetesFlex questionnaire** | **Actions to ensure all items are covered** |
| --- | --- | --- | --- |
| Background | Photography | Not covered | No actions planned |
| Background | Have you recently been to your GP? | Not covered |  |
| Background | HbA1c | Covered |  |
| Background | Diabetes duration | Not covered | Covered in the existing medical record checklist |
| Background | Insulin Time in Range (TIR)/Time Out of Range (TOR) | Partially covered with TIR |  |
| Background | Any annual consultation last 15 months | Not covered | Covered in the existing medical record checklist |
| Background | Comorbidities | Partially covered through items on blood pressure and cholesterol |  |
| Hypoglycemia | Number of hypoglycemic events per week | Covered with a four-week time frame |  |
| Hypoglycemia | How worried are you of getting hypos? | Covered |  |
| Hypoglycemia | Have you ever had a serious hypo? | Covered |  |
| Hypoglycemia | Can you feel if you are getting a hypo? | Covered |  |
| Late complications | Blood pressure | Covered in the open text fields if raised by the patients | Covered in the optional consultation questionnaire |
| Late complications | Cholesterol | Covered in the open text fields if raised by the patients | Covered in the optional consultation questionnaire |
| Late complications | Eye health and retinopathy | Covered |  |
| Late complications | Feet and neuropathy | Covered |  |
| Late complications | How well do you manage any late complications? | Partially covered |  |
| Late complications | Changes in late complications | Partially covered |  |
| Lifestyle | Smoking | Covered in the open text fields if raised by the patients |  |
| Needs | Need for prescription refill | Not specifically covered, the patient can raise this question in the open text fields | No actions planned |
| Needs | Open text field for the patient to describe what they want their next consultation to address | Covered |  |
| Needs | Do you have any need or whish for a consultation at this time? | Covered |  |
| Needs | What is the patient’s self-management goals | Covered |  |
| Needs | Which healthcare profession do the patient want to meet? Physician, nurse, dietitian, etc. | Covered |  |
| Needs | Self-management challenges | Covered |  |
| Needs | Encourage the patient to address own needs | Covered |  |
| Self-management | Infiltrates | Covered |  |
| Self-management | Do you experience hyperglycemic events in which you cannot find the cause? | Not specifically covered, the patient can raise this question in the open text fields |  |
| Self-management | Mood or irritability in relation to the blood glucose levels | Partially covered through items and through open text fields |  |
| Self-management | How do you experience your self-management? | Partially covered |  |
| Self-management | How do you experience your diabetes now? | Partially covered |  |
| Self-management | How do you manage to keep a stable blood glucose level? | Not covered |  |
| Self-management | Food and carbohydrate counting | Covered |  |
| Self-management | Experienced control over own diabetes | Covered |  |
| Self-management | Level of energy connected to changes in blood glucose levels | Not specifically covered, but can be interpreted based on time on range and items of general well-being |  |
| Self-management | Skills, competencies, and knowledge | Covered |  |
| Self-management | Sleep | Not covered | Under consideration |
| Social factors | social constraints due to diabetes | Covered in the open text fields if raised by the patients |  |
| Social factors | Social relations | Partially covered |  |
| Social factors | Any major life events | Covered in the open text fields if raised by the patients |  |
| Social factors | Status on driver’s license | Not covered | Covered in adapted version |
| Social factors | Pregnancy or planning pregnancy | Covered in the open text fields if raised by the patients | Under consideration |
| Social factors | Employments | Partially covered |  |
| Social factors | Family relations or responsibility for children | Not covered | Under consideration |
| Symptoms | Symptoms | Covered |  |
| Symptoms | Associations between medication/treatment and fatigue | Partially covered |  |
| Symptoms | Open text fields to address whether they experience symptoms or diabetes related discomfort | Covered in the open text fields if raised by the patients |  |
| Treatment | Proportion of basal and bolus | Covered in the open text fields if raised by the patients | Data can be downloaded from the patient equipment |
| Treatment | Have you uploaded any blood glucose readings the last week? | Covered |  |
| Treatment | Do you experience variation in your blood glucose in which you cannot find the cause? | Partially covered |  |
| Treatment | Injection technique | Covered |  |
| Treatment | Uploading blood glucose data | Not covered | Aiming to connect with Diasend Glooko |
| Treatment | Do you use any technical equipment such as CGM, insulin pump etc. in your diabetes self-management? | Covered |  |
| Treatment | Do you experience any challenges with any technical equipment such as CGM, insulin pump etc. | Covered in the open text fields if raised by the patients |  |
| Treatment | Type of treatment and insulin regime | Covered |  |
| Treatment | If you experience high variation in blood glucose levels, when are you experiences hypos or hypers? | Not covered |  |
| Treatment | Would you want a change in your insulin regime? | Covered in the open text fields if raised by the patients |  |
